# PREB inhibits the replication of prototype foamy virus by affecting its transcription

**DOI:** 10.1186/s12985-023-02211-y

**Published:** 2023-10-26

**Authors:** Junshi Zhang, Yali Xu, Chenchen Wang, Xiaopeng Tuo, Xingli Zhao, Wentao Qiao, Juan Tan

**Affiliations:** 1 Key Laboratory of Molecular Microbiology and Technology, Ministry of Education, College of Life Sciences, Nankai University, Tianjin, 300071 China; 2 Department of Hematology, Oncology Centrer, Tianjin Union Medical Center, No.190 Jieyuan Road, Hongqiao District, Tianjin, 300121 P. R. China

**Keywords:** Prototype foamy virus, PREB, Tas, Transcription

## Abstract

**Background:**

Foamy viruses (FVs) are unique nonpathogenic retroviruses, which remain latent in the host for a long time. Therefore, they may be safe, effective gene transfer vectors. In this study, were assessed FV–host cell interactions and the molecular mechanisms underlying FV latent infection.

**Methods:**

We used the prototype FV (PFV) to infect HT1080 cells and a PFV indicator cell line (PFVL) to measure virus titers. After 48 h of infection, the culture supernatant (i.e., cell-free PFV particles) and transfected cells (i.e., cell-associated PFV particles) were harvested and incubated with PFVL. After another 48 h, the luciferase activity was used to measure virus titers.

**Results:**

Through transcriptomics sequencing, we found that *PREB* mRNA expression was significantly upregulated. Moreover, *PREB* overexpression reduced PFV replication, whereas endogenous *PREB* knockdown increased PFV replication. PREB interacted with the Tas DNA-binding and transcriptional activation domains and interfered with its binding to the PFV long terminal repeat and internal promoter, preventing the recruitment of transcription factors and thereby inhibiting the transactivation function of Tas. PREB C-terminal 329–418 aa played a major role in inhibiting PFV replication; PREB also inhibited bovine FV replication. Therefore, PREB has a broad-spectrum inhibitory effect on FV replication.

**Conclusions:**

Our results demonstrated that PREB inhibits PFV replication by impeding its transcription.

**Supplementary Information:**

The online version contains supplementary material available at 10.1186/s12985-023-02211-y.

## Introduction

Foamy viruses (FVs; also known as spumaviruses) are unique and complex retroviruses [1]. In contrast to other retroviruses, FVs have two promoters in their genome: the long terminal repeat (LTR) and the internal promoter (IP) [2]. FVs are nonpathogenic and only maintain latent infection in the host [3]. The FV genome codes for not only structural proteins (Gag, Pol, and Env) but also nonstructural proteins (Tas and Bet) [4]. In prototype FV (PFV), Tas is called Bel-1 (between *env* and LTR), whereas it is called Borf1 (BFV open reading frame 1) or BTas (bovine Tas) in bovine FV (BFV). Tas is mainly involved in the regulation of viral gene expression, and it may also be important for persistent or lytic infection of FVs [5]. PFV Tas contains at least two functional domains: DNA-binding domain (DNA-BD) at the N-terminus and transcriptional activation domain (AD) at the C-terminus [6, 7].

As a transcriptional regulator of FVs, Tas protein plays a crucial role in virus replication. Some cell factors inhibit FV replication by interacting with Tas. For instance, promyelocytic leukemia (PML) binds to the N-terminal of PFV Tas to prevent it from recognizing viral promoters, thereby inhibiting Tas transactivation function [8]. The protein p53-induced RING-H2 (Pirh2) can interact with and degrade PFV Tas through the ubiquitin–proteasome pathway, thereby inhibiting PFV gene expression [9]. We previously found that interferon-induced protein 35 (IFP35) interacts with BTas to impede its recruitment of transcription factors and thereby inhibit BFV replication [10]. In contrast, N-Myc interactor (Nmi) inhibits PFV replication by interacting with Tas and then sequestering it in the cytoplasm [11]. We recently noted that serum/glucocorticoid regulated kinase 1 (SGK1) interacts with PFV Tas AD, impeding Tas-induced transactivation and affecting viral transcription [12].

Prolactin regulatory element binding (PREB), also known as Sect. 12 [13–15], is a transcription factor [length = 417 amino acids (aa)] ubiquitously expressed in many tissues [14]. PREB contains two potential transregulatory PQ-rich domains and 3 WD-repeat regions; thus, it is also considered a member of the eukaryotic WD-repeat protein family. Members of this protein family are involved in many cellular functions, including signal transduction, RNA processing, cytoskeleton assembly, vesicle trafficking, and gene regulation [16]. However, how PREB affects transcriptional regulation differs considerably from other members of the WD-repeat protein family because PREB can stimulate gene expression by directly binding to DNA [14, 17].

PREB plays a significant transcriptional regulatory role, upregulating the expression of some genes. For instance, PREB binds to and activates the prolactin promoter, upregulating prolactin expression [18]. PREB also mediates steroid 11β-hydroxylase (*CYP11B1*) transcription by binding to the *CYP11B1* promoter [19]; it also mediates scavenger receptor class B type I transcription by binding to the PREB response element of its promoter [20]. However, PREB can also downregulate the expression of some genes; for example, it negatively regulates the expression of the gluconeogenic gene by directly binding to the prolactin core binding element on their promoters, and it responds to cyclic AMP activation in adipocytes inhibiting adiponectin gene expression [21].

Few studies thus far have focused on PREB and viruses. Only one study demonstrated that PREB interacts with the hepatitis C virus (HCV) protein NS4B to bind to the HCV replication complex and then promotes HCV RNA replication by participating in the formation of the membranous replication compartment. Furthermore, HCV infection can induce PREB expression in vitro and in vivo [22]. However, studies on the interaction between PREB and retrovirus are lacking. Therefore, whether PREB plays a role in PFV infection warrants further exploration.

In this study, we assessed the effects of PREB on PFV transcription. Our results indicated that PREB, specifically PREB C-terminal 329–418 aa, impedes the function of Tas DNA-BD and AD, thereby inhibiting PFV replication.

## Materials and methods

### Plasmid constructs and antibodies

Human PREB cDNA was cloned into pCMV-3HA (Clonetech, Mountain View, CA, USA). PREB truncations were generated on the basis of pCMV-3HA-PREB. pLTR-luc [23], pIP-luc [23], and PFV full-length infectious clone (pcPFV) [24] were kindly provided by Maxine L. Linial (Division of Basic Sciences, Fred Hutchinson Cancer Research Center, Seattle, WA, USA). We purchased constructs of the pCMV-AD, pCMV-BD, and pFR-Luc from Strategene (La Jolla, CA, USA) and pCMV-β-Gal from Invitrogen (Carlsbad, CA, USA). The constructs of p3.1-Tas [25], pFlag-Tas [25], the BFV infectious clone pBS-BFV [26], pCMV-AD-Tas (1–220 aa), and pCMV-BD-Tas (223–300 aa) [12] were prepared as described previously. The sequences of all constructs were confirmed through sequencing.

We purchased antibodies against PREB from Proteintech (Hubei, China); antibodies against Flag and Myc from Sigma (St. Louis, MO, USA); antibodies against tubulin and HA and horseradish peroxidase (HRP)-conjugated secondary antibodies from Santa Cruz Biotechnology (Santa Cruz, CA, USA); and fluorescein isothiocyanate (FITC)-conjugated secondary antibodies from Jackson ImmunoResearch Laboratories. Finally, antibodies against PFV Gag [25] and BFV Gag [27] were prepared in our laboratory.

### Cell culture and transfection

HEK293T, HT1080, HeLa, PFV indicator cell line (PFVL), and BFV indicator cell line (BFVL) cells were cultured in Dulbecco’s modified Eagle’s medium containing 10% (v/v) fetal bovine serum at 37 °C under 5% CO_2_. Plasmid transfection was performed using polyethyleneimine (PEI; Polysciences, Warrington, PA, USA), according to the manufacturer’s instructions [23].

### PFV production and infection

HEK293T cells were transfected with 10 µg of pcPFV. After 48 h, the cells were centrifuged at 1000 g for 10 min, and the supernatants were collected and stored at 4 °C. The multiplicity of infection (MOI) was calculated according to the method of Tai et al. [28].

For virus infection, HT1080 cells were infected with PFV stock. After 48 h, the supernatants and 1/10 of the infected cells were collected and cocultured with PFVL cells. Next, luciferase activity was measured, and relative luciferase activation was used to indicate the virus titer. The remaining cells were analyzed through Western blotting.

### Knockdown cell line generation

The knockdown cell lines were screened using a retrovirus vector system. MLV Gag-pol (1 µg), VSV-G (0.5 µg), and shControl/shRNA (1 µg) plasmids were transfected into HEK293T cells. After 48 h, the cells were centrifuged at 1000 g for 10 min, and the supernatants were collected. Then, the pseudovirus in the supernatant was stored at − 80 °C. HT1080 cells were infected with the pseudovirus and then subcultured in a selection medium containing 2 µg/mL puromycin. Western blotting was used to detect the knockdown efficiency.

### Luciferase reporter assay

Cells were harvested 48 h after transfection or infection. Their luciferase activity was measured using a luciferase reporter assay system kit (Promega, Madison, WI, USA), according to the manufacturer’s instructions.

### Alu polymerase chain reaction

To detect the integration level of the virus, we transfected HT1080 cells with PREB and the empty vector. The experimental group was treated with reverse transcriptase inhibitor AZT (10 µM) [29] and integrase inhibitor raltegravir (10 µM) [30] before virus infection. After 2 h, PFV stock was added to infect the cells; after 30 h, the cells were collected, and total DNA was extracted using DNeasy Blood and Tissue Kit (Qiagen, Duesseldorf, Germany), according to the manufacturer’s instructions. Integrated proviral DNA was measured as described previously [25].

### Immunofluorescence assay

HeLa cells were added to coverslips and fixed with 4% paraformaldehyde in PBS at room temperature for 10 min; the cells were then permeabilized with 0.1% Triton X-100 in PBS for 10 min. After blocking for 2 h, the cells were incubated with anti-HA or anti-Flag for 2 h. The cells were then incubated with FITC or tetramethylrhodamine isothiocyanate (TRITC)-conjugated goat secondary antibodies for 45 min. Their nuclei were stained with 2.5 µg/mL DAPI for 10 min. Target protein localization was observed under an Olympus X71 fluorescence microscope.

### Coimmunoprecipitation

HEK293T cells were collected and lysed using an immunoprecipitation lysis buffer (50 mM Tris-HCL, pH 8.0, 150 mM NaCl, 1% NP-40, 50 × Cocktail). The lysates were incubated with the antibodies for 3 h and then exposed to Protein A–agarose at 4 °C for 3 h. This mixture was washed six times with the immunoprecipitation lysis buffer and then mixed with a loading buffer; next, it was boiled to 100 °C for Western blotting analysis.

### Chromatin immunoprecipitation assay

PFVL cells were transfected with 3HA-PREB, Flag-Tas, or Flag-Tas + 3HA-PREB; the transfected cells were collected and then their DNA was amplified through semiquantitative polymerase chain reaction to detect the amount of target DNA. The primer sequences were as follows: PFV LTR, 5′-GTGAGATCGAATCTTTCCTTAAC-3′ (forward) and 5′-CCG TACAATCTAGAAACTATCC-3′ (reverse); GADPH, 5′-TACTAGCGGTTTTACGGGCG-3′ (forward) and 5′-TCGAACAGGAGGAGCAGAGAGCGA-3′ (reverse).

Next, chromatin immunoprecipitation (ChIP) assay was performed using the EZ-chip kit (Millipore, Burlington, MA, USA), according to the manufacturer’s protocol. The assay was performed as described previously [12]. The amounts of Tas and PREB in the transfected cells were measured through Western blotting.

### Western blotting

Cells were collected, lysed using RIPA buffer, and then centrifuged at 12,000 g and 4 °C for 10 min. The supernatant was mixed with the loading buffer and then boiled to 100 °C for 15 min. The protein samples were then separated through sodium dodecyl sulfate-polyacrylamide gel electrophoresis and subsequently transferred onto polyvinylidene fluoride membranes. The membranes were blocked for 45 min; next, they were incubated with the primary antibodies for 1.5 h and then with horseradish peroxidase (HRP)-conjugated secondary antibodies for 45 min. The reacting bands were detected using an enhanced chemiluminescence system (Advansta, Menlo Park, CA, USA).

### Statistical analysis

Data are presented as means ± standard deviations (SDs) from the results of three independent experiments. Statistical analysis was performed using GraphPad Prism (version 8.0; GraphPad, San Diego, CA, USA). *P* < 0.05 was considered to indicate statistical significance (*P<0.05; **P<0.01; ***P<0.001; ****P<0.0001).

## Results

### PREB upregulation after PFV infection

**Fig. 1:**
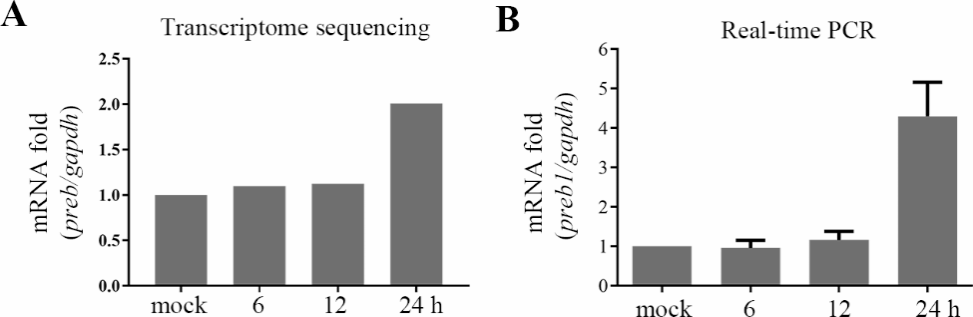
PREB upregulation after PFV infection. (**A**) The result of transcriptomics sequencing. (**B**) PFV (MOI = 0.5) infected HT1080 cells at 6 h, 12 h, 24 h. Total RNA was extracted and reverse transcribed into cDNA. Next, Real-time PCR was performed to detect the mRNA level of *PREB*

**Fig. 2:**
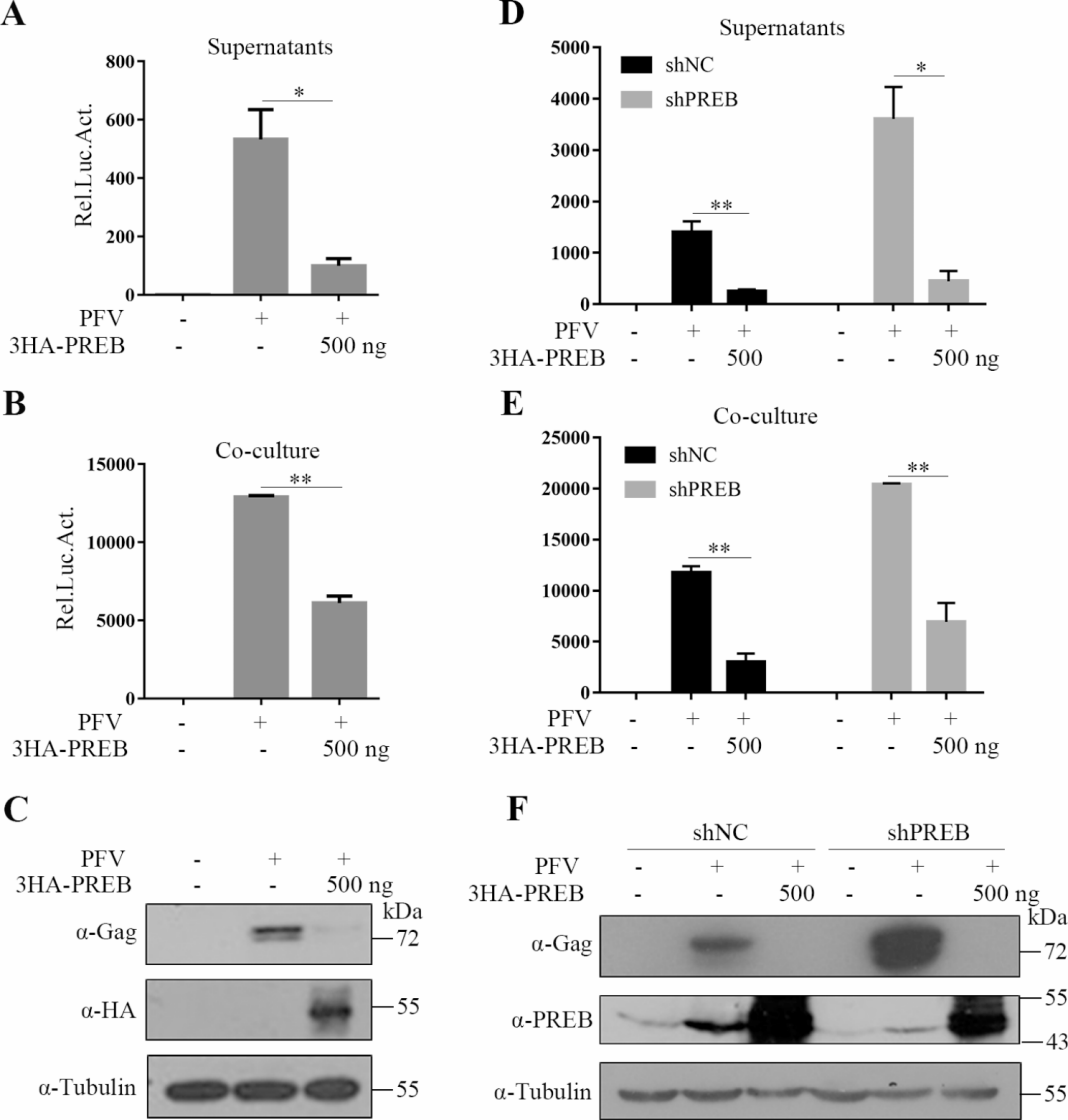
Inhibition of PFV replication by PREB. (**A**–**C**) HT1080 cells were infected with PFV (MOI = 0.5) after transfected with PREB or empty vector for 8 h. After 40 h, 600 µl of supernatants (**A**) or 1/10 infected HT1080 cells (**B**) were co-cultured with PFVL cells to determine viral titers by luciferase assay. (**C**) Western blotting was used to detect viral protein expression level. (**D**–**F**) PFV (MOI = 0.5) infected HT1080-shControl and HT1080-shPREB cells. After 8 h, HT1080 cells were transfected PREB or empty vector. After 48 h, 600 µl of supernatants (**D**) or 1/10 infected HT1080 cells (**E**) were co-cultured with PFVL cells, the luciferase activity was measured 48 h later. (**F**) The rest HT1080 cells were lysed for Western blot analysis. *P<0.05, **P<0.01

To identify the host factors affecting PFV replication, we used a PFV stock to infect HT1080 cells, followed by transcriptome sequencing. The results demonstrated significant alterations in mRNA levels of 394 genes in PFV-infected cells after 24 h [12]. In particular, *PREB* mRNA expression was significantly upregulated after PFV infection (Fig. 1A); thus far, few studies have focused on PREB–PFV interactions. Therefore, the effects of PREB on PFV replication subsequently. When confirming the transcriptome sequencing results, we found that *PREB* mRNA expression was significantly upregulated after PFV infection (Fig. 1B). When exploring the effect of endogenous PREB on PFV replication, we found that PFV infection upregulates PREB levels (Fig. 2F, lanes 1 and 2).

### Inhibition of PFV replication by PREB

Next, we cotransfected PREB and its empty vector into HT1080 cells. After 8 h, PFV stocks were used to infect these cells. Compared with control cells, PREB-overexpressing cells significantly reduced both cell-free (Fig. 2A) and cell-associated PFV (Fig. 2B) levels. Moreover, it significantly reduced PFV Gag expression in transfected cells (Fig. 2C). These results confirmed that PREB overexpression inhibits the replication of PFV.

To explore whether endogenous PREB also inhibits PFV replication, we infected PREB-knockdown or control HT1080 cells with a PFV stock. As shown in Fig. 2D–F, PFV replication levels were significantly higher in the knockdown cells than in the control cells, indicating that endogenous PREB had an inhibitory effect on PFV replication. To further confirm the inhibitory effect of PREB on PFV replication, we reinduced PREB expression in PREB-knockdown and control cells and found that PREB re-expression led to downregulation of PFV replication, confirming the inhibitory effect of PREB on PFV replication.

### Inhibition of Tas-mediated transactivation of PFV LTR and IP by PREB

**Fig. 3:**
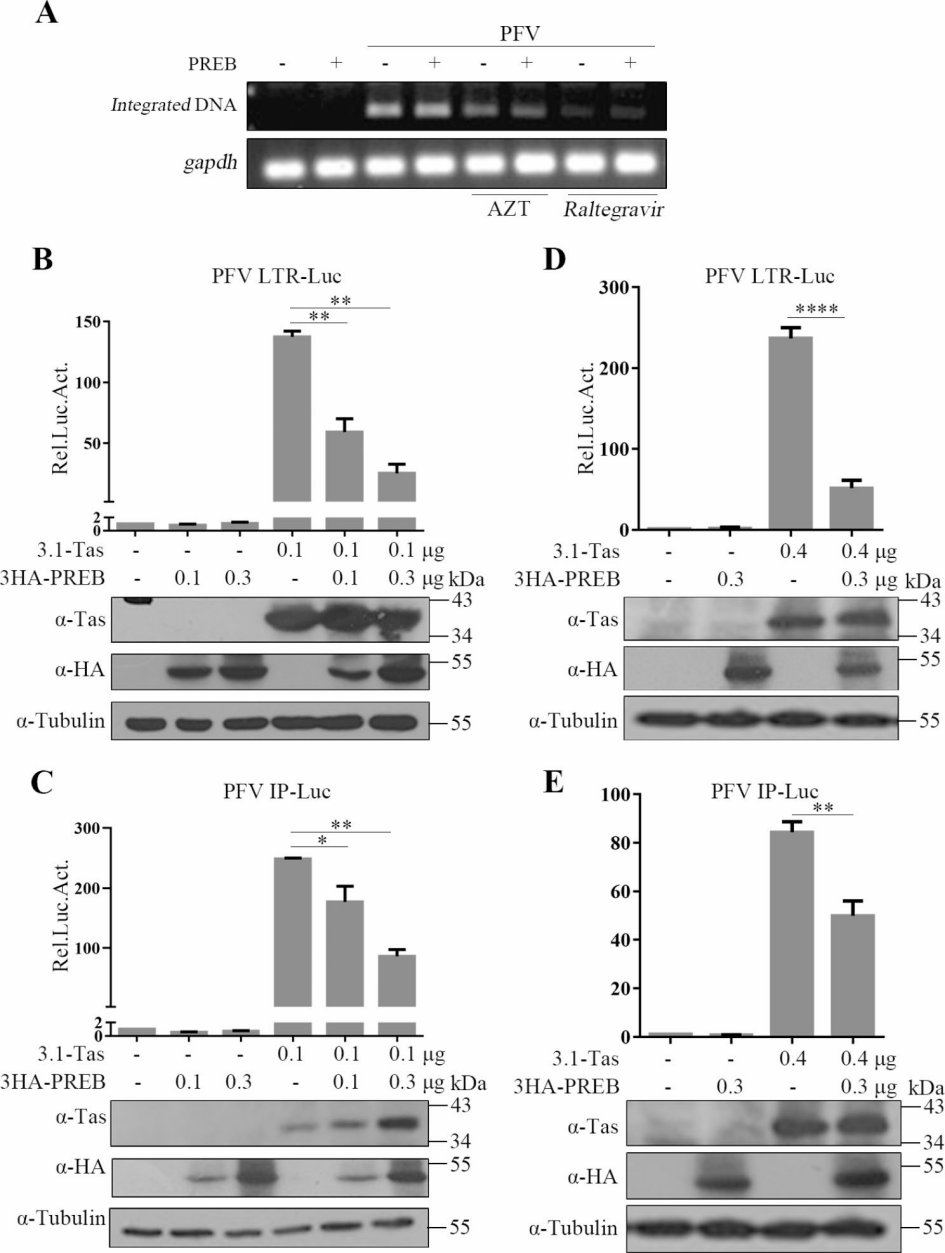
Inhibition of Tas-mediated transactivation of PFV LTR and IP by PREB. (**A**) Integrated proviral DNA was measured as described in Materials and Methods. (**B** and **C**) HEK293T cells were transfected with PFV LTR-Luc (**B**) or IP-Luc (**C**) and pCMV-β-gal, combined with Tas and increasing amount of PREB. After 48 h, luciferase activity was measured. The remaining cell lysates were collected for Western blot analysis. (**D** and **E**) HT1080 cells were transfected with PFV LTR-Luc (**D**) or IP-Luc (**E**) and pCMV-β-gal, combined with Tas and PREB. After 48 h, luciferase activity was measured. The remaining cell lysates were collected for Western blot analysis. * *P*<0.05, ** *P*<0.01, **** *P*<0.0001

Next, we explored the specific mechanism underlying inhibition of PFV replication by PREB. Genome integration is considered the boundary between early and late retrovirus replication. Therefore, we assessed whether PREB affects PFV genome integration through Alu polymerase chain reach [31]. As shown in Fig. 3A, treatment with raltegravir (integrase inhibitor) and AZT (reverse transcriptase inhibitor) significantly inhibited PFV genome integration. However, PREB overexpression had no significant effect on PFV genome integration. In other words, PREB could not affect the early replication stage of PFV.

In the PFV lifecycle, genome integration is followed by transcription. Therefore, we next analyzed whether PREB affects PFV transcription. Because transcription in PFVs is mediated by Tas, we used PFV LTR-Luc and IP-Luc reporter plasmids to explore the effects of PREB on the basic transcriptional activity of PFV promoters and the transactivation ability of Tas. As shown in Fig. 3B and C, PREB overexpression had no significant effect on the basic transcriptional activity of PFV LTR and IP; nevertheless, it inhibited Tas-mediated transactivation of PFV LTR and IP in a dose-dependent manner. Identical results were obtained in HT1080 cells (Fig. 3D and E).

### Inhibition of PFV replication by PREB 329–418 aa

**Fig. 4:**
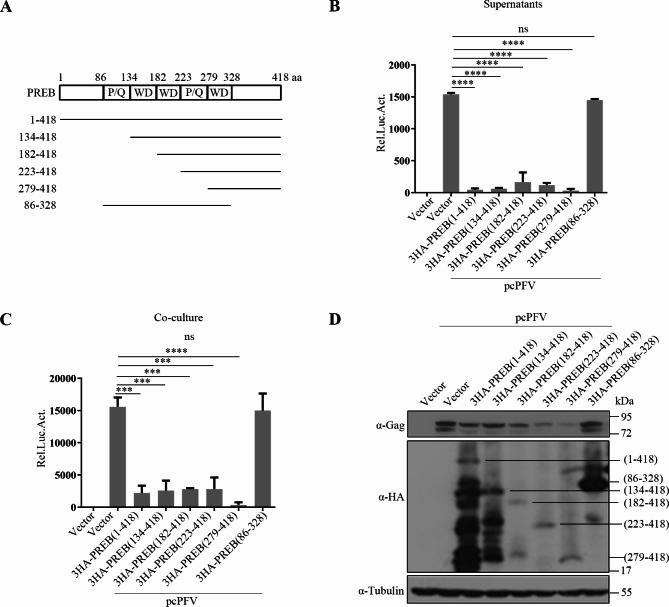
Inhibition of PFV replication by PREB 329–418 aa. (**A**) Truncations of PREB protein. (**B**–**D**) HEK293T cells were transfected with pcPFV, the empty vector or PREB and different truncations. After 48 h, culture supernatants (600 µl) (**B**) or 1/20 transfected HEK293T cells (**C**) were co-cultured with PFVL cells. Luciferase activity was measured 48 h post-infection. (**D**) The rest HEK293T cells were lysed for Western blot analysis. *** *P*<0.001, **** *P*<0.0001 and ns for *P* > 0.05

Next, we explored the key PREB domains playing an antiviral role. Five truncates were constructed according to the PREB structure (Fig. 4A). These truncated expression plasmids or empty vectors were cotransfected with the full-length PFV construct (pcPFV) into HEK293T cells. As shown in Fig. 4B–D, overexpression of 3HA-PREB, 3HA-PREB 134–418 aa, 3HA-PREB 182–418 aa, 3HA-PREB 223–418 aa, and 3HA-PREB 279–418 aa significantly inhibited PFV replication. However, 3HA-PREB 86–328 aa overexpression led to no inhibition of PFV replication. Therefore, the key domain of PREB that inhibits PFV replication must be PREB C-terminal 329–418 aa. Notably, we observed a small difference in the results of Western blotting for Gag levels and those of the viral infectivity determined using luciferase assay, which may be due to the higher sensitivity of luciferase assay.

### Inhibition of PFV transcription by PREB 329–418 aa

**Fig. 5:**
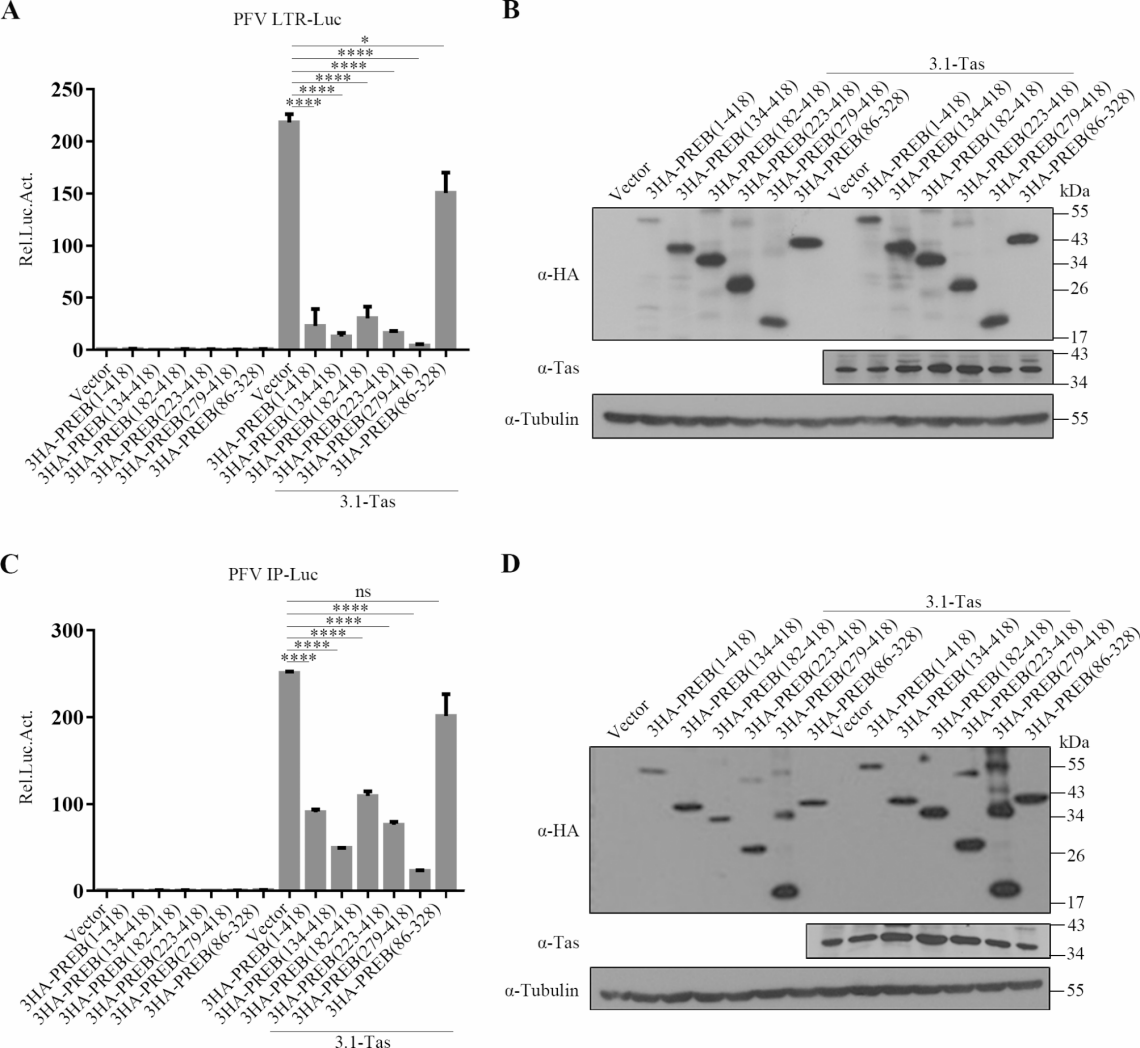
Inhibition of PFV transcription by PREB 329–418 aa. (**A** and **B**) HEK293T cells were transfected with PREB or truncated plasmids, LTR-Luc and pCMV-β-gal, combined with 3.1-Tas or the empty vector. After 48 h, luciferase activity was measured (**A**). The remaining cell lysates were collected for Western blot analysis (**B**). (**C** and **D**) HEK293T cells were transfected with PREB or truncated plasmids, IP-Luc and pCMV-β-gal, combined with 3.1-Tas or the empty vector. After 48 h, luciferase activity was measured (**C**). The remaining cell lysates were collected for Western blot analysis (**D**). * *P*<0.05, ****P<0.0001 and ns for *P* > 0.05

We also explored the key domains of PREB inhibiting transcription in PFVs. As shown in Fig. 5A–D, overexpression of 3HA-PREB, 3HA-PREB 134–418 aa, 3HA-PREB 182–418 aa, 3HA-PREB 223–418 aa, and 3HA-PREB 279–418 aa significantly inhibited Tas transactivation of PFV LTR and IP, but 3HA-PREB 86–328 aa overexpression led to no inhibition of Tas-mediated transactivation. These results indicated that PREB inhibits the transcription of PFV through its C-terminal 329–418 aa, consistent with its results for PFV replication inhibition.

### PREB–Tas interactions

**Fig. 6:**
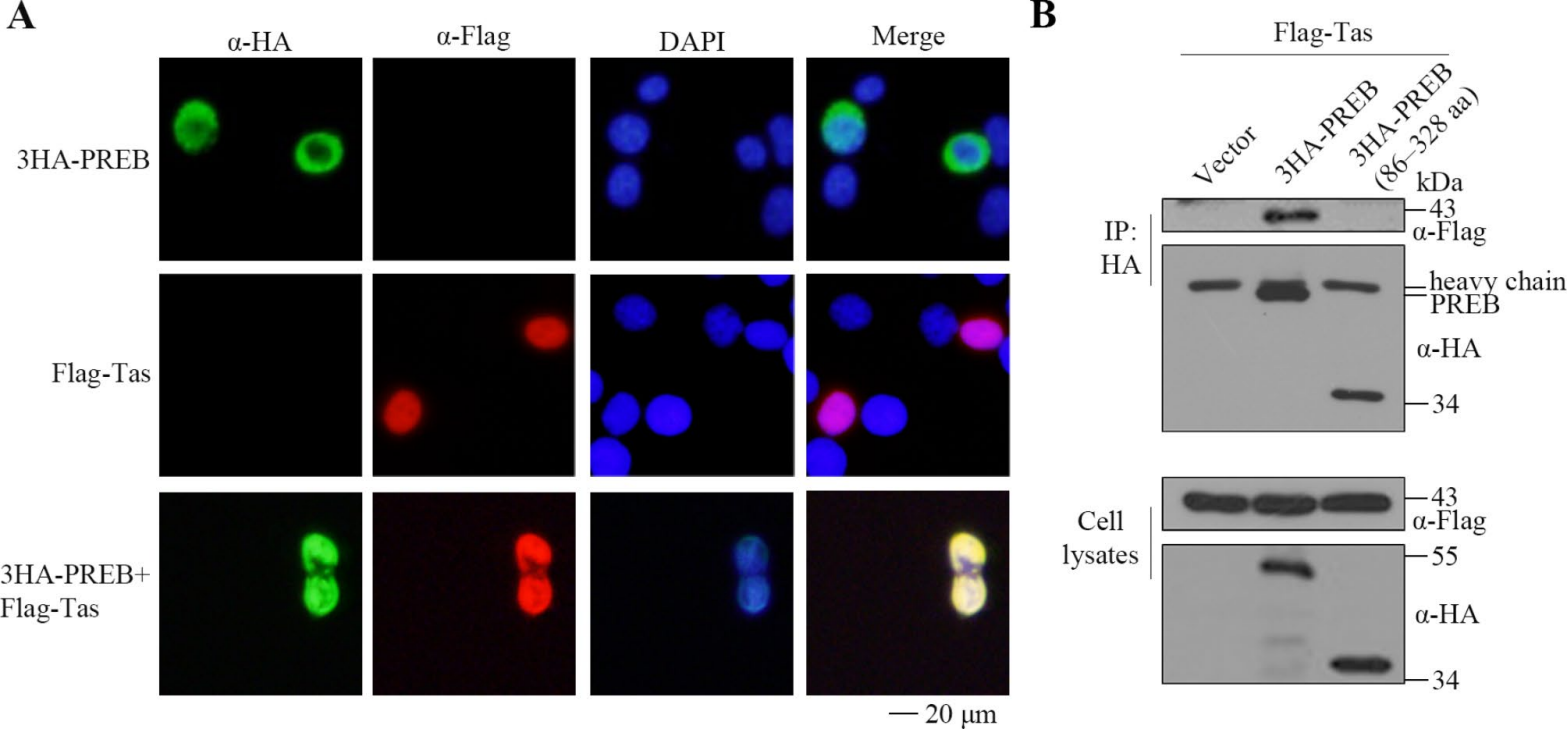
PREB–Tas interactions. (**A**) HeLa cells were transfected with 3HA-PREB or Flag-Tas or both, indirect immunofluorescence was used to localize PREB and Tas. (**B**) Flag-Tas and 3HA-PREB, 3HA-PREB (86–328 aa) or empty vector were co-transfected into HEK293T cells. After 48 h, co-immunoprecipitation was performed with HA antibodies. Western blot analysis of samples from cell lysates and immunoprecipitates using HA and Flag antibodies

Because Tas must enter the nucleus to perform its function, we explored whether PREB affects nuclear localization of Tas. Our immunofluorescence assay results demonstrated that PREB was located in the nucleus and cytoplasm, whereas Tas was located only in the nucleus. When PREB and Tas were coexpressed, they were both located only in the nucleus (Fig. 6A). Moreover, PREB 86–328 aa was located in the cytoplasm, but it did not affect the nuclear localization of Tas (Fig. S1A). Our coimmunoprecipitation assay results further confirmed the interaction of PREB or PREB 86–328 aa with Tas. As shown in Fig. 6B and S1B and C, PREB interacted with Tas, but the interaction between PREB 86–328 aa and Tas was almost nonexistent. Therefore, we speculate that PREB interacts with Tas through its N-terminal 1–85 aa and C-terminal 329–418 aa to affect the transactivation function of Tas, thereby inhibiting PFV replication.

### Effects of PREB 329–418 aa on PFV Tas DNA-BD and AD function

**Fig. 7:**
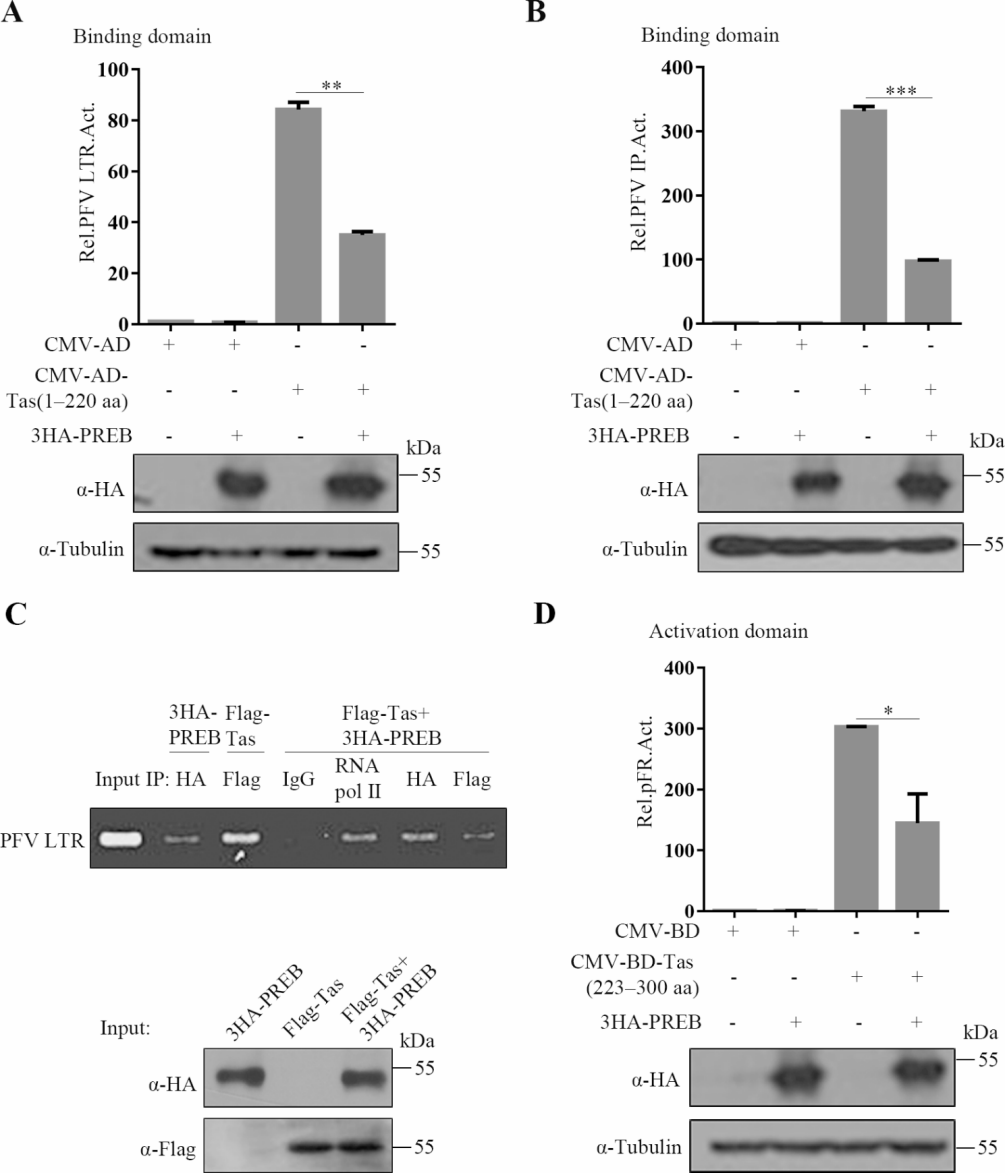
Effects of PREB 329–418 aa on PFV Tas DNA-BD and AD function. (**A** and **B**) HEK293T cells were transfected with pCMV-AD or pCMV-AD-Tas (1–220 aa), 3HA-PREB, LTR-Luc (**A**) or IP-Luc (**B**) and pCMV-β-gal. After 48 h, luciferase activity was measured. (**C**) PFVL cells were co-transfected with 3HA-PREB, Flag-Tas, Flag-Tas + 3HA-PREB, respectively. At 48 h post-transfection, cells were subjected to a ChIP assay as described in Materials and Methods. Subsequently, PCR amplification was carried out to detect LTR in the immunoprecipitated chromatin fragments. (**D**) HEK293T cells were transfected with pCMV-BD or pCMV-BD-Tas (223–300 aa), 3HA-PREB, pFR-Luc and pCMV-β-gal. After 48 h, luciferase activity was measured. * *P*<0.05, ** *P*<0.01, *** *P*<0.001

Next, we investigated the mechanism by which PREB inhibits PFV transcription. By using the mammalian one hybrid system [12], we investigated whether PREB affects the function of the Tas DNA-BD and AD and noted that PREB inhibited Tas binding to PFV LTR and IP (Fig. 7A and B). Our ChIP assay results also confirmed that PREB impedes the binding of Tas to PFV LTR (Fig. 7C), which further shows that PREB affects the DNA-binding function of Tas. We further explored whether PREB affects the transcriptional activation function of Tas and found that PREB inhibited Tas AD function (Fig. 7D). To clarify whether PREB affects Tas function by interacting with its DNA-BD and AD, we performed coimmunoprecipitation and noted that PREB interacted with full-length Tas as well as its DNA-BD and AD (Fig. S2). Thus, PREB may impede the function of these two structural domains through interaction.

To further clarify whether PREB affects the function of Tas through its C-terminal 329–418 aa and thus inhibits PFV replication, we used the mammalian one-hybrid assay on PREB and its truncated plasmids (encoding PREB 1–328 aa, PREB 86–418 aa, and PREB 86–328 aa; Fig. S3A). As shown in Fig. S3B and C, both 3HA-PREB and 3HA-PREB 86–418 aa affected the function of Tas DNA-BD and AD, but 3HA-PREB 1–328 aa and 3HA-PREB 86–328 aa did not affect these two structural domains. These results demonstrated that C-terminal 329–418 aa is the key structural domain of PREB inhibiting PFV replication.

### Inhibition of BFV replication by PREB

Next, we explored whether the antiviral effect of human PREB is specific to PFV. We assessed the effects of PREB in BFV, which also belongs to the Spumaretrovirnae subfamily and is part of the broad antiviral spectrum of PREB. As shown in Fig. S4A and B, PREB overexpression significantly reduced the BFV titer, as well as cell BFV Gag expression. Thus, PREB inhibit BFV replication.

We further explored the molecular mechanism underlying the inhibition of BFV by PREB. Because PREB inhibits PFV replication by inhibiting PFV transcription, we first explored the effects of PREB on BFV transcription. As shown in Fig. S4C and D, PREB did not affect the basic transcriptional activity of BFV LTR and IP, but it significantly inhibited the transactivation of BFV LTR and IP by BTas. Therefore, PREB also inhibit BFV replication by acting on the transcriptional step.

## Discussion

Few studies thus far have analyzed the effects of host genes on FV replication. To further explore the mechanisms underlying FVs’ latent infection and analyze their interaction with hosts, we infected HT1080 cells with PFVs and then performed transcriptome sequencing (GSE200199). The results demonstrated that *PREB* mRNA expression was upregulated significantly, suggesting that PREB may participate in FV replication. In the subsequent experiments, we confirmed that PFV infection upregulates *PREB* mRNA expression; moreover, our virus infection experiment results indicated that PFV infection can upregulate endogenous PREB expression (Fig. 2F). *PREB* overexpression could significantly inhibit PFV replication and endogenous *PREB* knockdown enhances PFV replication. Furthermore, PREB re-expression inhibited PFV replication significantly. Taken together, these results demonstrated that PREB inhibits PFV replication. Most proteins previously noted to inhibit FV replication are interferon-induced proteins, such as TRIM5α [32], APOBEC3G [33], PML [8], IFP35 [10], Nmi [11], SLFN11 [34], Tetherin [35], PHF11 [36] and IFITM3 [37]. Recently, some non–interferon-inducible proteins, such as Pirh2 [9], TBC1D16 [38], and SGK1 [12], were noted to inhibit PFV replication. In the current study, we noted that PREB, also a non–interferon-inducible protein, also inhibits FV replication.

We previously confirmed that PFV infection enhances SGK1 promoter activity via Tas, resulting in upregulation of endogenous *SGK1* mRNA and protein expression [12]. In the current study, we noted that PFV infection upregulates *PREB* mRNA and protein expression. Considering that PFV Tas can upregulate the expression of certain cellular genes [39], further investigation confirming whether PFV upregulates PREB expression through Tas is warranted. PREB interacts with Tas through its N-terminal 1–85 aa and C-terminal 329–418 aa, interfering with Tas-mediated transactivation of PFV LTR and IP and thereby inhibiting PFV replication. Similarly, host factors such as PML [8], Nmi [11], IFP35 [10], Trim28 [40], Pirh2 [9], and SGK1 [12] have been noted to all interact with Tas and then impeding its function. Only a few studies have assessed the function of N- and C-terminal subunits of PREB, and their specific role in antiviral function warrants further exploration.

As a transcription regulator, PREB can directly bind to DNA to regulate gene expression and upregulate or downregulate the expression of some genes [14, 41]. Our current results revealed that PREB does not affect PFV Tas expression; this is unlike Pirh2, which affects Tas transactivation function by interacting with Tas and downregulating its expression [42]. Moreover, in contrast to PML (only affecting the function of Tas DNA-BD) [8] or IFP35 and SGK1 (only affecting the function of Tas AD) [10, 12], we found that PREB affects the function of both the Tas DNA-BD and AD. Our sequence analysis results revealed the presence of potential PREB core binding element TGAT in Tas DNA-BD of PFV LTR [14, 18]. Our ChIP assay results also demonstrated that PREB can bind to PFV LTR and that it affects the binding ability of Tas. These results suggest that PREB competes with Tas for binding to PFV LTR, thereby affecting the transactivation function of Tas. Moreover, PREB affects the function of Tas AD by interacting with Tas and influencing its transcription factor recruitment.

We also noted that PREB can not only inhibit PFV but also effectively inhibit BFV replication by inhibiting BTas-mediated transcription of BFV LTR and IP, indicating PREB’s broad-spectrum retrovirus inhibitory activity. Most studies on PREB have focused on its function as a transcription factor, which regulates gene expression. In PREB–virus interactions, PREB is a novel HCV host cofactor. Kong et al. reported that PREB is induced by HCV infection and recruited into the replication complex through interaction with NS4B and that recruited PREB promotes HCV RNA replication by participating in membranous HCV replication compartment formation [22]. In the current study, we, for the first time, confirmed the effects of PREB on retroviruses, demonstrating the protein’s antiviral spectrum.

## Conclusions

Our study first demonstrated that PFV infection upregulates PREB expression and that PREB inhibits PFV replication. We also identified the mechanisms underlying PFV replication inhibition by PREB and the key domains involved in the process. The current results enhance the understanding of PREB function and indicate the broad scope of FV–host interactions.

### Electronic supplementary material

Below is the link to the electronic supplementary material.

Supplementary Material 1

Supplementary Material 2

Supplementary Material 3

Supplementary Material 4

## Data Availability

The sequencing data were deposited at the Gene Expression Omnibus (GEO) repository (accession number is GSE200199).

## Abbreviations

FVs: Foamy viruses
PFV: Prototype foamy virus
BFV: Bovine foamy virus
Bel-1: Between *env* and LTR
Borf1: BFV open reading frame 1
BTas: Bovine Tas
PML: Promyelocytic leukemia
Pirh2: p53-induced RING-H2 protein
IFP35: Interferon-induced protein 35
Nmi: N-Myc interactor
SGK1: Serum/glucocorticoid regulated kinase 1
PREB: Prolactin regulatory element binding
PCBE: Prolactin core binding element
HCV: Hepatitis C virus
PFVL: PFV indicator cell line
