# Supplementary material for: PREB inhibits the replication of prototype foamy virus by affecting its transcription
Source: Virol J. 2023 Oct 26;20:244. doi: 10.1186/s12985-023-02211-y (PMC10604407; doi:10.1186/s12985-023-02211-y)
Supplement: Supplementary file 1 — Supplementary Material 1 [file 12985_2023_2211_MOESM1_ESM.docx]

**Fig.S1**

**
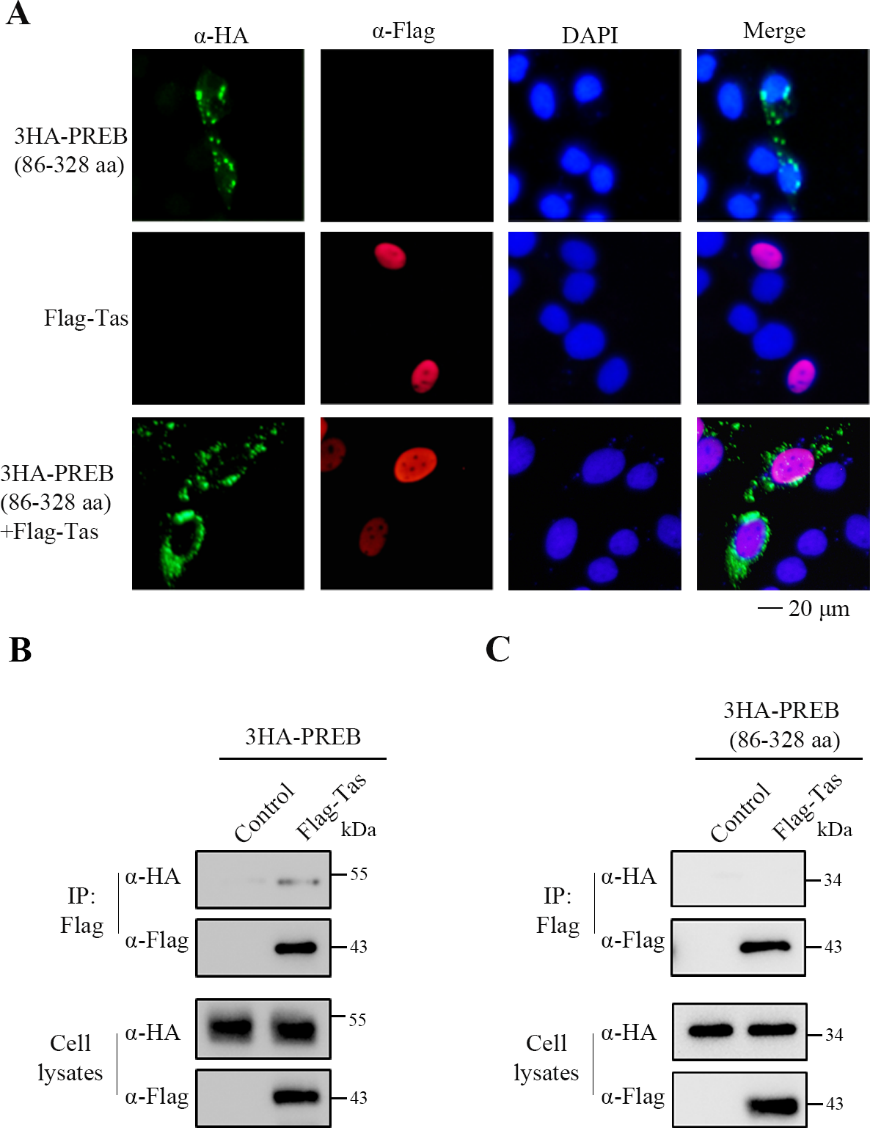
**

**Fig.S1.** PREB–Tas interactions. (**A**) HeLa cells were transfected with 3HA-PREB (86-328 aa) or Flag-Tas or both, indirect immunofluorescence was used to localize PREB (86-328 aa) and Tas. (**B**) 3HA-PREB and Flag-Tas or empty vector were co-transfected into HEK293T cells. After 48 h, co-immunoprecipitation was performed with Flag antibodies. Western blot analysis of samples from cell lysates and immunoprecipitates using HA and Flag antibodies. (**C**) 3HA-PREB (86-328 aa) and Flag-Tas or empty vector were co-transfected into HEK293T cells. After 48 h, co-immunoprecipitation was performed with Flag antibodies. Western blot analysis of samples from cell lysates and immunoprecipitates using HA and Flag antibodies.
