# Supplementary material for: PREB inhibits the replication of prototype foamy virus by affecting its transcription
Source: Virol J. 2023 Oct 26;20:244. doi: 10.1186/s12985-023-02211-y (PMC10604407; doi:10.1186/s12985-023-02211-y)
Supplement: Supplementary file 2 — Supplementary Material 2 [file 12985_2023_2211_MOESM2_ESM.docx]

**Fig.S2**

**
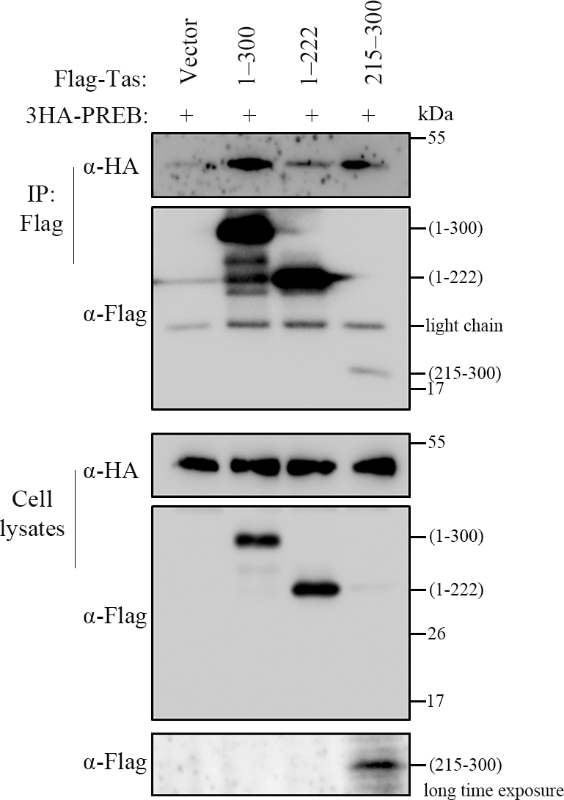
**

**Fig.S2.** PREB–Tas interactions. 3HA-PREB and Flag-Tas or its truncated were co-transfected into HEK293T cells. After 48 h, co-immunoprecipitation was performed with Flag antibodies. Western blot analysis of samples from cell lysates and immunoprecipitates using HA and Flag antibodies.
