# Supplementary material for: PREB inhibits the replication of prototype foamy virus by affecting its transcription
Source: Virol J. 2023 Oct 26;20:244. doi: 10.1186/s12985-023-02211-y (PMC10604407; doi:10.1186/s12985-023-02211-y)
Supplement: Supplementary file 3 — Supplementary Material 3 [file 12985_2023_2211_MOESM3_ESM.docx]

**Fig.S3**

**
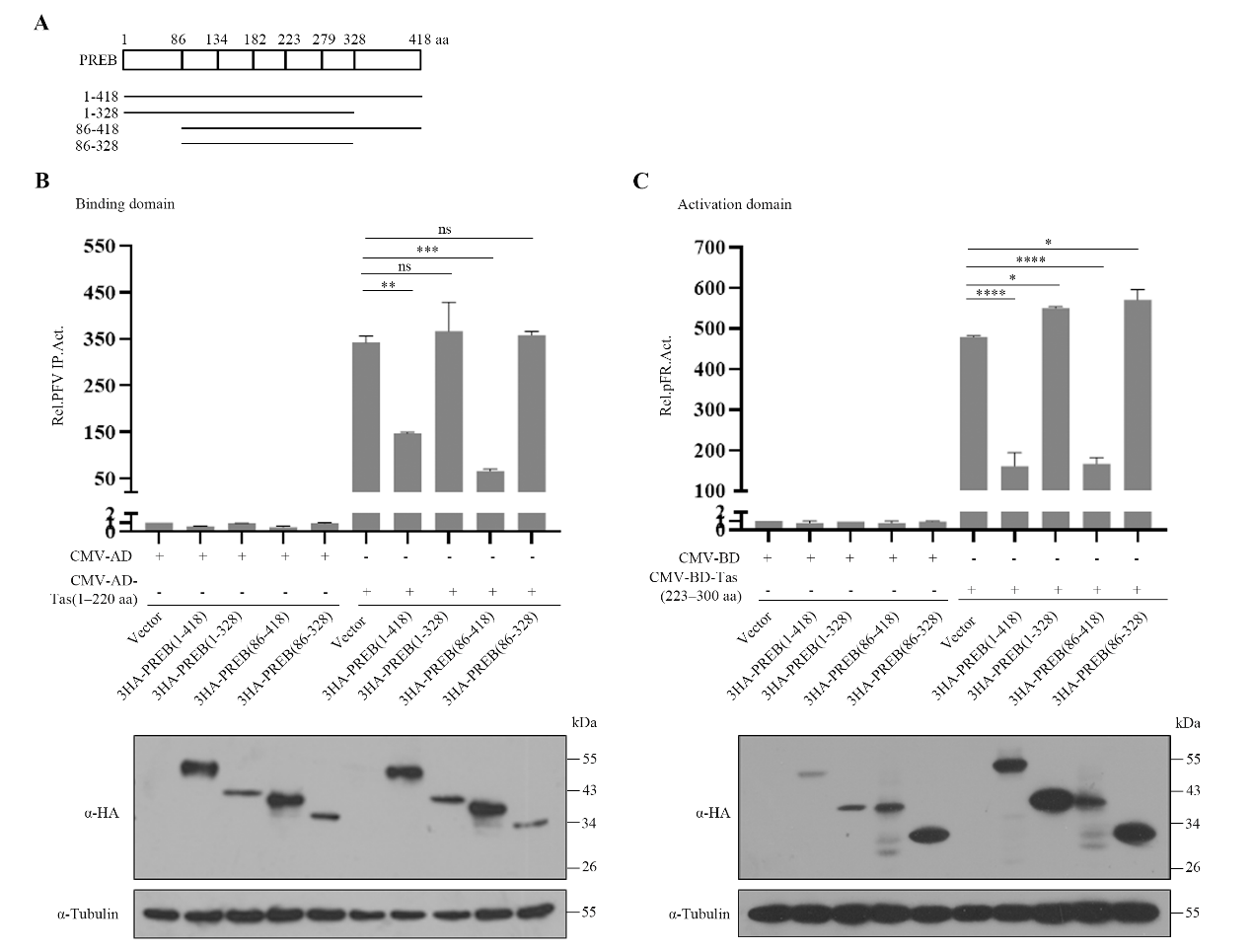
**

**Fig.S3.** Effects of PREB 329-418 aa on PFV Tas DNA-BD and AD function. (**A**) Truncations of PREB protein. (**B**) HEK293T cells were transfected with pCMV-AD or pCMV-AD-Tas (1–220 aa) (0.3 μg), 3HA-PREB or its truncated plasmids (0.3 μg), IP-Luc (0.025 μg) and pCMV-β-gal (0.025 μg). After 48 h transfection, luciferase activity was measured. (**C**) HEK293T cells were transfected with pCMV-BD or pCMV-BD-Tas (223–300 aa) (0.02 μg), 3HA-PREB or its truncated plasmids (0.03 μg), pFR-Luc (0.01 μg) and pCMV-β-gal (0.025 μg). After 48 h transfection, luciferase activity was measured. * *P*＜0.05, ** *P*＜0.01, *** *P*＜0.001, *****P*＜0.0001 and ns for *P* > 0.05.
