# Supplementary material for: PREB inhibits the replication of prototype foamy virus by affecting its transcription
Source: Virol J. 2023 Oct 26;20:244. doi: 10.1186/s12985-023-02211-y (PMC10604407; doi:10.1186/s12985-023-02211-y)
Supplement: Supplementary file 4 — Supplementary Material 4 [file 12985_2023_2211_MOESM4_ESM.docx]

**Fig.S4**

**
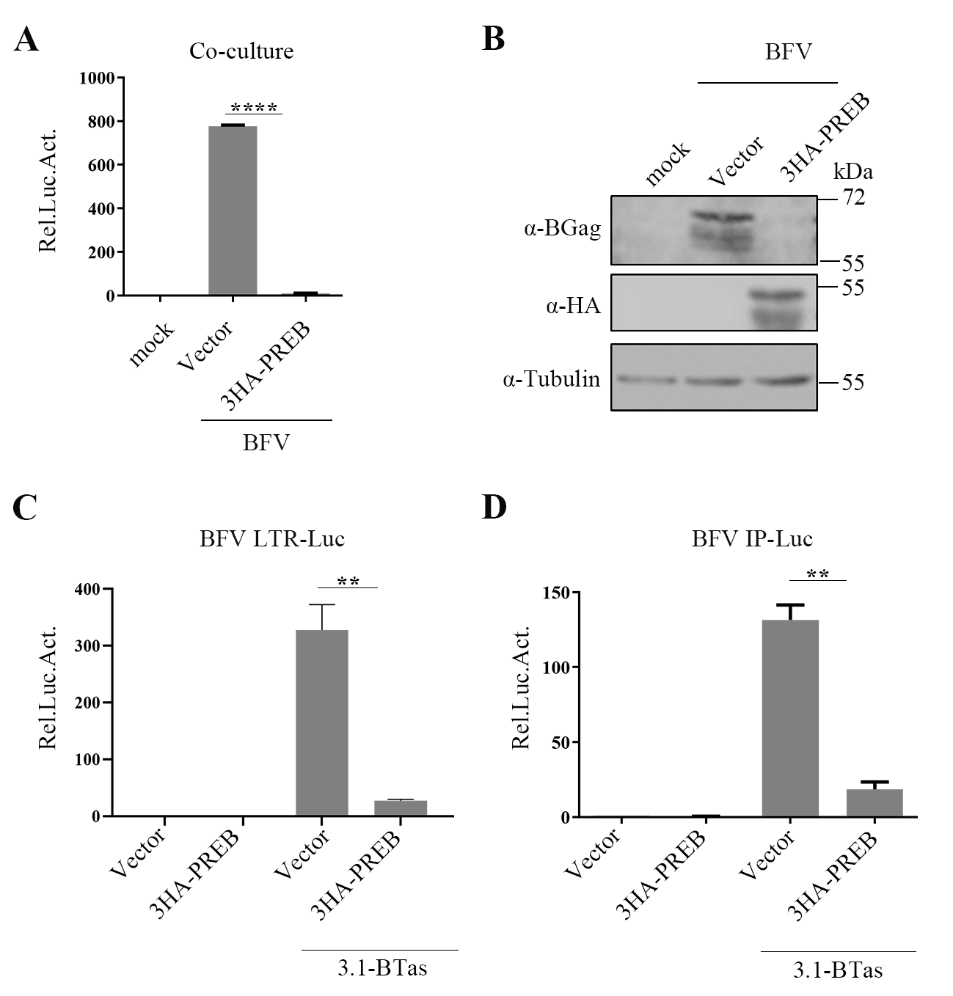
**

**Fig.S4.** Inhibition of BFV replication by PREB. (**A**) HEK293T cells were transfected with BFV and the empty vector or PREB. After 48 h, 1/20 of transfected HEK293T cells were co-cultured with BFVL cells. Luciferase activity was measured 48 h post-infection. (**B**) The rest HEK293T cells were lysed for Western blot analysis. (**C** and **D**) HEK293T cells were transfected with 3HA-PREB, BFV LTR-Luc (**C**) or IP-Luc (**D**), and pCMV-β-gal, combined with 3.1-BTas or the empty vector. After 48 h, luciferase activity was measured. ** *P*＜0.01, **** *P*＜0.0001.
